# Exercise training promotes neurogenesis in the adult hippocampus with a particular focus on cell cycle regulation

**DOI:** 10.3389/fspor.2026.1770350

**Published:** 2026-06-23

**Authors:** Xiao-Tao Wang, Jia Wang, Xiao-Jiao Zhao

**Affiliations:** 1 Hainan Police College, Haikou, Hainan, China; 2 School of Clinical Medicine & Laboratory Medicine, Jiangsu University, Zhenjiang, Province, China; 3 Department of Basic Teaching, Liaoning Police College, Dalian, Liaoning, China

**Keywords:** cell cycle, exercise, hippocampus, neural stem cells, neurogenesis, self-renewal

## Abstract

Adult neurogenesis is predominantly restricted to two neurogenic regions in the mammalian brain: the subventricular zone (SVZ) of the lateral ventricle and the subgranular zone (SGZ) of the dentate gyrus (DG) within the hippocampus. The hippocampus serves as a critical brain structure involved in learning and memory processes, and the continuous generation of new neurons contributes to enhanced synaptic plasticity. Accumulating evidence has demonstrated that impaired hippocampal neurogenesis is closely associated with various neuropsychiatric disorders, including Alzheimer's disease, epilepsy, and traumatic brain injury. Although the precise molecular and cellular mechanisms underlying adult neurogenesis remain incompletely elucidated, extensive research over the past several decades has identified numerous endogenous, exogenous, and environmental factors that modulate this process. Notably, exercise training, as a key exogenous stimulus, has been shown to promote adult hippocampal neurogenesis by influencing the neurochemical environment and functional integration of newly generated neurons. This review aims to summarize the relationship between cell cycle dynamics and adult hippocampal neurogenesis, with a particular emphasis on how physical exercise regulates the cell cycle to activate and promote the proliferation of neural stem cells (NSCs) in the DG, thereby facilitating the differentiation and lineage progression of neural progenitor cells. A deeper understanding of the regulatory mechanisms by which exercise enhances adult hippocampal neurogenesis may provide novel insights into the development of therapeutic strategies for neurological and psychiatric disorders.

## 1 Introduction

Since Altman first proposed the existence of new neurons in the adult brain in 1,965 numerous studies have been conducted to investigate the cellular and molecular mechanisms underlying adult neurogenesis (1, 2). This process originates from a population of radial glial-like precursor cells (type 1 cells) that have astrocytic properties, express marker of neural stem cells (3, 4). Adult neurogenesis is predominantly restricted to two neurogenic regions in the brain—the subventricular zone (SVZ) of the lateral ventricle and the subgranular zone (SGZ) of the dentate gyrus (DG) (5).

Adult neurogenesis is a highly dynamic biological process that can be negatively influenced by stress and aging, while being positively modulated by physical activity (such as running and swimming) (6), enriched environmental stimuli (7), and cognitive learning experiences (8). Various neurological conditions, including brain injury (9), neurodegenerative diseases (e.g., Alzheimer's disease (10) and Parkinson's disease (11), as well as the natural process of aging (12), are frequently associated with diminished neurogenic capacity. As a non-invasive and low-risk intervention with significant neurogenic benefits, exercise training has garnered increasing interest among neuroscientists for its potential therapeutic applications (13).

Numerous studies have demonstrated that voluntary exercise training represents one of the most potent stimulators of adult neurogenesis (14–16). As extensively documented, exercise induces neurogenic activity in both canonical neurogenic niches—the subgranular zone (SGZ) of the hippocampus and the subventricular zone (SVZ) (17). however, the underlying mechanisms are regionally distinct: in the SVZ, lactate-mediated activation of the hydroxycarboxylic acid receptor 1 (HCA1) has been identified as a key driver of exercise-induced neurogenesis, whereas such a lactate–HCA1 axis has not been demonstrated in the hippocampal SGZ (17). In this review, we first summarize the relationship between cell cycle dynamics and adult hippocampal neurogenesis. Subsequently, we focus on the mechanisms by which exercise training modulates each stage of the neurogenic lineage progression to promote adult neurogenesis.

## 2 Research progress on cell cycle dynamics and adult hippocampal neurogenesis

Adult neurogenesis in the DG of the hippocampus is a multi-stage developmental process. The majority of NSCs in the DG remain in a quiescent state under normal conditions and rarely enter the cell cycle (18). Upon activation, these quiescent NSCs transition into an activated state and re-enter the cell cycle. Activated NSCs subsequently undergo asymmetric division to produce intermediate progenitor cells (type 2) and neuroblasts (type 3), progressing through these sequential stages to ultimately differentiate into immature neurons and post-mitotic mature neurons (18). Newly generated neurons originating from the SGZ migrate into the inner granule cell layer of the dentate gyrus and fully differentiate into glutamatergic excitatory dentate granule cells (19).

Among these stages, the transition of NSCs from a quiescent state to an activated state is considered the most critical step in adult hippocampal neurogenesis (20). Chiani and colleagues have demonstrated that WAF1/cyclin-dependent kinase inhibitor 1 (p21^Cip1/Waf1^) plays a pivotal role in regulating the transition of NSCs from quiescence to activation (21). Under physiological conditions, the cell cycle inhibitor p21 maintains NSCs in the G_0_ phase of the cell cycle, thereby preserving the NSC pool in the DG of the hippocampus (21). In response to brain injury, NSCs deficient in p21 exit the quiescent state (G_0_→G_1_ phase) and proceed through asymmetric division to differentiate into post-mitotic neurons, thereby replacing neurons lost due to injury. However, prolonged activation and proliferation of NSCs may ultimately result in the depletion of the NSC pool (21).

In recent years, neurobiologists have employed transgenic mouse models expressing cell cycle proteins to conclusively demonstrate that aberrant cell cycle regulation represents a key factor influencing adult hippocampal neurogenesis (22). Based on these findings, the “cell cycle length hypothesis” has been proposed. This hypothesis posits that dysregulation of key G1-phase regulators—including cyclin-dependent kinase 6 (Cdk6) (23) and the cyclin-dependent kinase inhibitor p21 (10)—alters the kinetics of the G1-to-S phase transition, thereby coordinately modulating neural progenitor proliferation, cell fate commitment, and neuronal differentiation within the adult hippocampus. The research team led by Wang et al. demonstrated that the transcriptional regulatory factor FOXG1 counteracts p21-mediated cell cycle arrest, leading to a shortened cell cycle duration in NSCs and an expansion of the neural precursor cell pool (10, 24). This mechanism contributes to the alleviation of impaired adult neurogenesis in Alzheimer's disease (AD) (10). Collectively, these findings indicate that the activation and fate specification of adult neural stem cells are tightly regulated by the expression and function of specific cell cycle proteins.

## 3 Research Status of exercise training regulation on adult hippocampal neurogenesis

In recent years, numerous studies have demonstrated that exercise training can enhance cell proliferation in the DG of the hippocampus and significantly increase the number of newly generated neurons in rodent models (19). Accumulating evidence indicates that physical activity not only stimulates adult hippocampal neurogenesis but also facilitates the functional integration of these newly formed neurons into existing neural circuits, thereby enhancing synaptic plasticity (25, 26). These findings are supported by observations of improved learning and memory performance in rats, along with a significant increase in long-term potentiation (LTP) (25). Moreover, exercise training has been shown to delay or partially mitigate the progression of neurodegenerative diseases (27), restore neurogenic capacity in HIV-infected mice (28), and ameliorate cognition and learning deficits following Alzheimer's disease (29). Additionally, exercise effectively promotes adult hippocampal neurogenesis and alleviates behavioral abnormalities associated with various neurological and psychiatric disorders (15, 25). Nevertheless, the precise mechanisms underlying these effects remain a topic of ongoing investigation within the field of neurobiology.

## 4 Research progress on the regulation of the cell cycle by exercise training in promoting adult hippocampal neurogenesis

### 4.1 Exercise training enhances the activation of NSCs

New neurons are generated within the adult hippocampal neurogenic niche from NSCs located in the DG (20). A subset of NSCs possesses the capacity to transition between quiescent and mitotically active states (30). Exercise training has been shown to activate quiescent NSC populations (20). Dong et al. administered the thymidine analog 5-ethynyl-2'-deoxyuridine (EdU) to transgenic mice expressing nestin and observed that prolonged activation of granule cells in the DG during voluntary exercise training induced the activation of quiescent NSCs in the subgranular zone, ultimately leading to the generation of new neurons (31).

### 4.2 Exercise training enhances the proliferation of neural progenitor cells

We have previously outlined that the expansion of the neural progenitor cell pool is closely associated with adult hippocampal neurogenesis (10, 24, 32). Recent studies have demonstrated that voluntary running selectively reduces the duration of the G_1_-S phase (from 12.9 h to 10.2 h) and the entire cell cycle (G_1_-S-G_2_-M) (from 24.9 h to 22.0 h), thereby stimulating the proliferation of neural progenitor cells in the DG of the hippocampus (33). Farioli-Vecchioli et al. have demonstrated that exercise training not only modulates cell cycle kinetics and supports hippocampal neurogenesis under physiological conditions, but also confers therapeutic beneficial in mice with genetic deficiencies in cell cycle regulatory proteins—such as the anti-proliferative protein BTG1 (33), which leads to the rescue of behavioral deficits, including impairments in pattern separation, by promoting the proliferation, differentiation, and integration of new neurons derived from NSCs (33). Notably, due to the postnatal deficiency of the cell cycle inhibitor gene *Btg1*, NSCs in these knockout mice exhibit excessive proliferation, which leads to the rapid depletion of the NSC pool in the DG and subsequent impairment of neurogenesis (33). However, a 12-day exercise training regimen significantly enhanced neural progenitor cell proliferation and long-term neurogenesis in the DG of these p21 knockout mice (33). This effect was primarily mediated by the activation of quiescent NSCs and the shortening of the G_1_-S phase in both NSCs and intermediate progenitor cells, enabling them to rapidly pass through the G_1_-S restriction point and complete the proliferation process (33). Battistini using p21 knockout mice that lacked the ability to exhibit cell cycle arrest, revealed that exercise training significantly enhances neuroregeneration following traumatic brain injury, as evidenced by: (i) increased numbers of neuroblasts; (ii) enhanced migration of newly generated neurons toward the injured cortical area; (iii) greater differentiation of new neurons in the perilesional region; and (iv) improved functional recovery at multiple time points after injury (34). These findings indicate that running training can significantly reduce the S phase duration of neuroblasts and promote endogenous neuroregeneration after traumatic brain injury (34).

### 4.3 Exercise training enhances the differentiation and survival of dentate gyrus granule neurons

**Figure 1:**
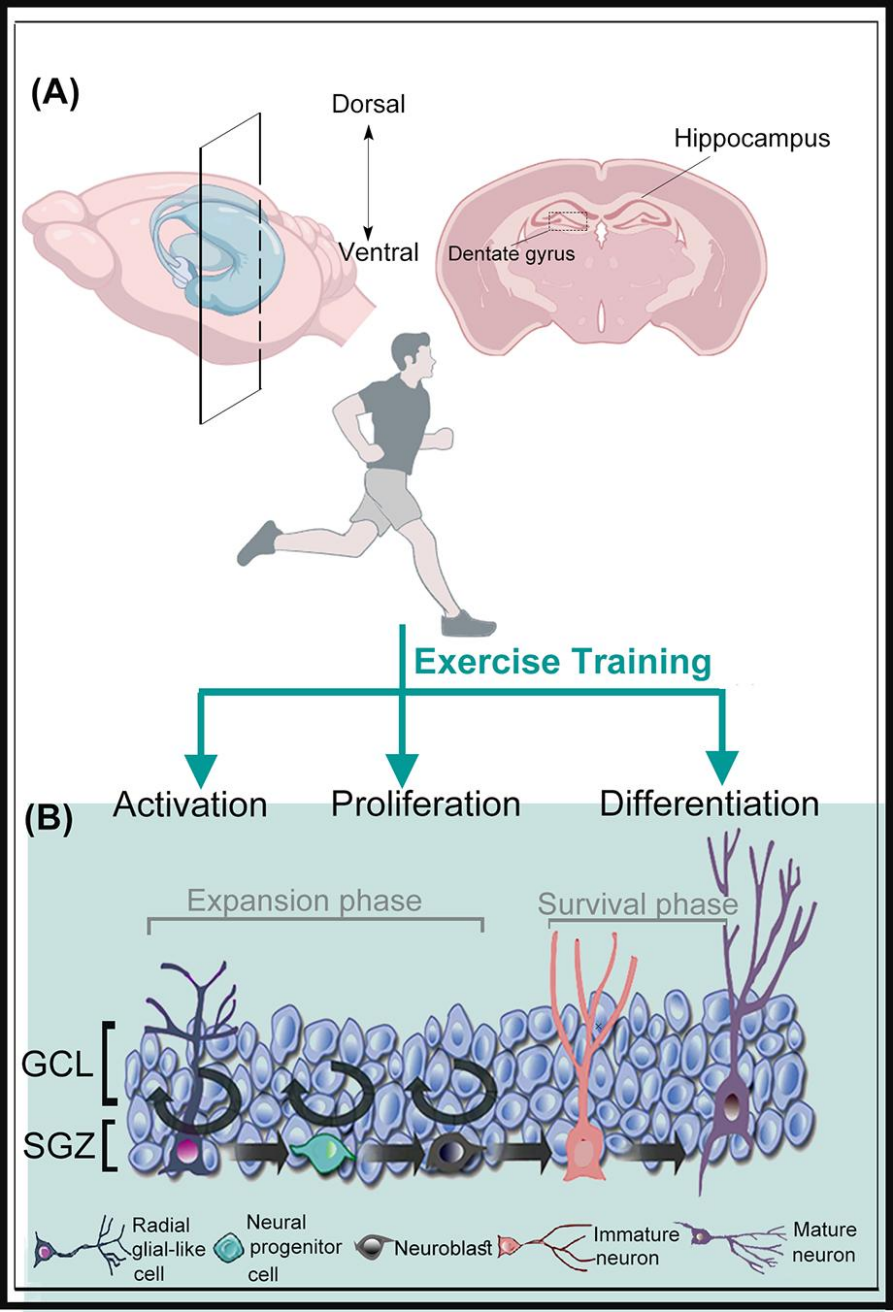
Exercise training enhances adult hippocampal neurogenesis. **(A)** Schematic representation of the mouse hippocampus. The panel illustrates a coronal cut through the dorsal hippocampus of a mouse brain. **(B)** Schematic representation of adult hippocampal neurogenesis. Exercise training activates quiescent NSCs that lead to the generation of daughter cells that differentiate into neurons. Neural progenitors that survive beyond the early survival stage mature into granule neurons and integrate into hippocampal circuitry.

In addition to promoting the proliferation of neural progenitor cells, exercise training has been demonstrated to enhance the differentiation and survival of newly generated neurons (35, 36). The van Praag research group investigated the effects of exercise training on neuronal differentiation in both young and aged mice, employing the mature neuron marker NeuN and the proliferation marker BrdU to assess newly formed DG neurons (37). Their findings revealed that although the proportion of newly generated neurons in aged mice was significantly lower compared to that in young mice (based on the total number of BrdU-labeled cells), both young and aged mice subjected to exercise training exhibited a significantly higher proportion of new neurons than their sedentary age-matched controls (37). Another study analyzed rats six days after BrdU administration and reported a marked increase in the proportion of BrdU and doublecortin (DCX)-labeled cells in the exercise-trained group (38). The elevated proportion of BrdU-positive cells expressing NeuN or DCX may reflect enhanced neuronal differentiation, improved survival of newborn neurons, or a combination of both mechanisms (39). These observations suggest that exercise training contributes to the differentiation and maturation of newly generated neurons in the adult hippocampus (40) (Figure 1).

### 4.4 Exercise training enhances the development and maturation of newly generated neurons

In addition to increasing the number of newly generated neurons, exercise training also influences their morphological development, particularly promoting the growth of axons and dendrites (16, 40). In a separate study, retroviruses expressing fluorescent proteins were employed to label dividing neural stem/progenitor cells, allowing for a comparative analysis of neuronal morphology between exercising and non-exercising mice (41). Compared to sedentary controls, exercising mice exhibited increased dendritic length and spine density in newly generated neurons (41). Voluntary exercise has been shown to accelerate the morphological maturation of new neurons—an observation that has been corroborated by other studies utilizing similar retroviral labeling techniques (42).

### 4.5 Exercise training enhances the functional integration of newborn neurons into hippocampal neural circuits

Newborn neurons must integrate with the existing hippocampal neural circuits to fulfill their functional roles (43). Therefore, assessing the synaptic connectivity of these newly generated hippocampal neurons is essential for elucidating the functional significance of adult neurogenesis. Using a virus-mediated monosynaptic retrograde tracing technique, Vivar et al. demonstrated that adult-born DG neurons initially establish local connections before forming distant presynaptic inputs (44). Although exercise training does not significantly alter the number of presynaptic neurons projecting to new neurons, it may modulate the properties of excitatory presynaptic inputs by influencing the integration of newly matured neurons into the circuitry (45). In other words, exercise training substantially affects the integration of adult-born neurons into hippocampal brain circuits.

## 5 Molecular mechanisms of exercise-induced neurogenesis in the adult hippocampus

Through candidate gene approaches and genome-wide gene expression analyses, neurobiologists have identified that physical exercise can induce the upregulation of various growth factors in the brains of animals, with brain-derived neurotrophic factor (BDNF) being a key mediator (46). Exercise training is believed to promote adult neurogenesis, at least in part, through the regulation of the neurotrophic factor BDNF (46). *In vivo* studies have demonstrated that the deletion of the TrkB gene, which encodes the high-affinity receptor for BDNF, attenuates neurogenesis and impairs the enhancement of LTP (47). Conversely, intracerebral administration of BDNF, which mimics the biochemical effects of exercise, has been shown to enhance neurogenesis in the adult hippocampus (48, 49).

Physical exercise can also induce the expression of several other growth factors, including nerve growth factor (NGF), which regulates cell proliferation, survival, and differentiation (50); fibroblast growth factor-2 (FGF-2), which modulates NF-kB expression (50); insulin-like growth factor 1 (IGF-1), which may exert anti-apoptotic effects (50); and vascular endothelial growth factor (VEGF), which promotes angiogenesis in the brain (51, 52). Additionally, a variety of hormones and peripheral signaling molecules have been identified as potential mediators of the neurogenic effects induced by physical activity. These include adiponectin leptin, angiotensin II, epinephrine, reactive oxygen species, AMP-activated protein kinase (AMPK), peroxisome proliferator-activated receptor (PPAR), PGC-1*α*, ciliary neurotrophic factor (CNTF), and pro-inflammatory cytokines (53–56). Given that these growth and trophic factors can be produced by multiple tissue types and various cell populations, their precise *in vivo* sources remain incompletely characterized. Moreover, their functional roles may vary depending on the temporal context and specific cellular environment (53–56). Consequently, the direct administration of these factors as potential therapeutic strategies for neurological disorders targeting the adult hippocampus remains a complex and challenging endeavor.

Another study has demonstrated that bone morphogenetic protein (BMP), functioning as a negative regulator of adult neurogenesis, plays a significant role in mediating the effects of exercise on hippocampal neurogenesis and hippocampus-dependent cognitive functions, including learning and memory (57). Exercise training enhances hippocampal neurogenesis and ameliorates hippocampus-associated cognitive and memory impairments by reducing BMP levels in the hippocampus (58). In contrast, overexpression of BMP4 inhibits exercise-induced cell proliferation and the generation of DCX-positive immature neurons (57). This observation aligns with previous findings indicating that activation of the BMP/BMPR1A signaling pathway in the hippocampus contributes to maintaining NSCs in a quiescent state (58). Exercise training may also stimulate the proliferation of hippocampal progenitor cells by modulating a key Wnt signaling inhibitor—secreted frizzled-related protein 3 (Sfrp3) (59). Sfrp3 is an inhibitory niche factor secreted by mature granule neurons in the local dentate gyrus and is highly expressed in adult dentate gyrus granule neurons as a Wnt antagonist (59). It regulates multiple stages of adult hippocampal neurogenesis. Genetic deletion of Sfrp3 has been shown to activate quiescent radial NSCs and enhance the maturation of newborn neurons, dendritic development, and spine formation in the adult mouse hippocampus (59). Furthermore, downregulation of Sfrp3 is essential for activity-dependent proliferation of adult neural progenitor cells and the maturation of newly generated neurons (59). Additionally, in investigating the cellular mechanisms underlying exercise-induced hippocampal neurogenesis, the Bartlett laboratory utilized Csf1r-GFP transgenic mice and found that the increased activity of neural precursor cells induced by exercise can be mediated by endogenous microglia (60). Through both *in vivo* and *in vitro* experiments, it was revealed that endogenous microglia exert bidirectional regulatory effects on the activation of neural precursor cells in the hippocampal dentate gyrus via the CX3CL1–CX3CR1 signaling axis (60).

Unbiased gene expression profiling analysis serves as a powerful experimental approach for comprehensively investigating the molecular mechanisms underlying exercise training-induced hippocampal neurogenesis. Early studies utilized tissue microarray techniques to examine the effects of exercise training on gene expression changes specifically in the DG region of the hippocampus. Tong L.Q. and colleagues conducted advanced high-density oligonucleotide microarray analysis using 5,139 gene probes to identify differentially expressed transcripts in the hippocampus of rats following three weeks of exercise training (61). Their results demonstrated that, compared to the sedentary control group, 88 genes in the exercise group exhibited expression changes exceeding 1.5-fold, with 44 genes upregulated and 44 downregulated (61). Pathway analysis further revealed that these differentially expressed genes were associated with neuronal activity, synaptic architecture, and neural plasticity (61). In studies exploring the relationship between exercise and aging, it has been shown that voluntary physical activity can mitigate the detrimental effects of aging on adult hippocampal neurogenesis (62). To elucidate the impact and underlying mechanisms of exercise training on hippocampal neurogenesis in aging, Stranahan et al. employed a highly sensitive Illumina bead microarray to assess the expression levels of over 24,000 genes in the hippocampus of aged mice (16 months old) that either engaged in voluntary running or remained sedentary (63). The findings indicated that upregulated genes were primarily involved in synaptic plasticity and mitochondrial function, whereas downregulated genes were associated with oxidative stress and lipid metabolism pathways (63).

To address how aerobic and non-aerobic fitness training differentially modulates brain structure and function, Griffin et al. (48) conducted a foundational study examining the effects of these two exercise paradigms on hippocampal-dependent learning and memory, as well as on serum BDNF levels (48). Their results demonstrated that both modalities promote adult hippocampal neurogenesis; however, acute high-intensity exercise elicited significantly greater neurogenic and cognitive enhancements—indicating that exercise intensity, rather than modality *per se*, is a primary determinant of neurobiological outcomes. Consistent with this interpretation, Lambertus et al. (17) reported that high-intensity interval training (HIIT) robustly enhances hippocampal neurogenesis to a greater extent than moderate-intensity continuous training (17). Furthermore, Voss et al. (64) employed functional magnetic resonance imaging (fMRI) to characterize exercise-induced functional plasticity in the aging brain and found that sustained aerobic training is associated with attenuated age-related decline in functional connectivity within higher-level cognitive networks (64). Collectively, these findings underscore that although physical exercise broadly benefits brain health, paradigms involving higher intensity and longer duration confer more robust enhancements to adult hippocampal neurogenesis and associated structural plasticity.

## 6 Conclusion

In summary, neurogenesis in the adult hippocampus is a persistent biological process. Exercise training has been shown to promote hippocampal neurogenesis in the DG, through multiple mechanisms including modulating the quiescence and activation of NSCs, enhancing the proliferation of neural progenitor cells, facilitating the development and maturation of newborn neurons, and integrating these neurons into existing hippocampal neural circuits. Accordingly, exercise has been shown to ameliorate cognitive and memory deficits in animal models of brain injury as well as in clinical populations with acquired brain injury. Therefore, exercise training holds significant potential for the clinical management and rehabilitation of patients with neurological injuries. Moreover, it offers considerable value in the treatment of individuals with neuropsychiatric disorders by reducing drug-related side effects, improving overall quality of life, and supporting functional recovery.
